# Amniotic fluid content in children with kidney and urinary tract anomalies determines pre- and postnatal development

**DOI:** 10.1007/s00467-023-05988-w

**Published:** 2023-05-23

**Authors:** Anne Mareike Schulz, Angela Lauten, Thomas Lehmann, Hans Proquitté, Felicitas Eckoldt, Friederike Weigel, Hans-Joachim Mentzel, Uwe Schneider, Ulrike John-Kroegel

**Affiliations:** 1 Department of Pediatrics, Pediatric Nephrology, University Hospital Jena, Am Klinikum 1, 07747 Jena, Thuringia Germany; 2 Department of Obstetrics and Gynecology, University Hospital Jena, Jena, Thuringia Germany; 3 Institute of Medical Statistics and Computer Science, University Jena, Jena, Thuringia Germany; 4 Department of Pediatrics, Section of Neonatology, University Hospital Jena, Jena, Thuringia Germany; 5 Department of Pediatric Surgery, University Hospital Jena, Jena, Thuringia Germany; 6 Institute of Diagnostic and Interventional Radiology, Section of Pediatric Radiology, University Hospital Jena, Jena, Thuringia Germany

**Keywords:** Renal oligohydramnios, Amniotic fluid volume, Amniotic fluid index, Antenatal counseling, Kidney and urinary tract anomalies

## Abstract

**Background:**

Renal oligohydramnios (ROH) describes an abnormally low volume of amniotic fluid (AF) during pregnancy. ROH is mostly caused by congenital fetal kidney anomalies. The ROH diagnosis frequently implies an increased risk of peri- and postnatal fetal mortality and morbidity. The present study aimed to evaluate the impact of ROH on pre-and postnatal development in children with congenital kidney anomalies.

**Methods:**

This retrospective study included 168 fetuses with anomalies in the kidney and urinary tract. Based on the amount of AF measured by ultrasound, patients were divided into three groups: normal amniotic fluid (NAF), amniotic fluid in the lower normal range (LAF), and ROH. These groups were compared with respect to prenatal sonographic parameters, perinatal outcomes, and postnatal outcomes.

**Results:**

Among the 168 patients with congenital kidney anomalies, 26 (15%) had ROH, 132 (79%) had NAF, and 10 (6%) had LAF. Of the 26 families affected by ROH, 14 (54%) decided to terminate pregnancy. Of 10 live-born children in the ROH group, 6 (60%) survived the observation time; of these, 5/6 presented with chronic kidney disease, stages I–III, at their last examination. The main differences in postnatal development between the ROH group and the NAF and LAF groups were: restricted height and weight gain, respiratory issues, complicated feeding, and the presence of extrarenal malformations.

**Conclusions:**

ROH is not a mandatory indicator of severe postnatal kidney function impairment. However, children with ROH have complicated peri-and postnatal periods, due to the presence of concomitant malformations, which must be considered in prenatal care.

**Graphical abstract:**

**Figure:**
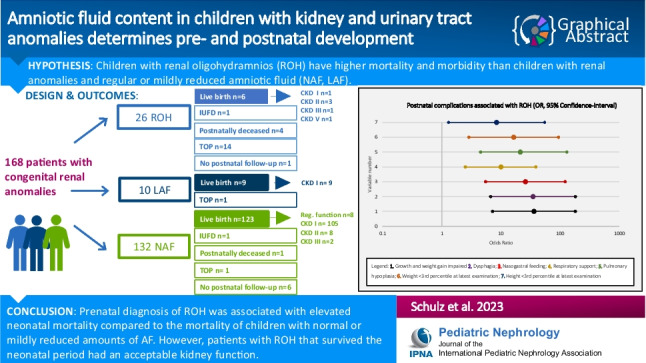
A higher resolution version of the Graphical abstract is available as Supplementary information

**Supplementary Information:**

The online version contains supplementary material available at 10.1007/s00467-023-05988-w.

## Introduction

An evaluation of amniotic fluid (AF) volume plays a major role in the assessment of fetal kidney function. Beginning in the 16^th^ week of gestation, fetal diuresis is responsible for more than half of the amount of AF. Hence, a malfunction in fetal urine production rapidly leads to renal oligohydramnios (ROH) [1]. The most common pathologies associated with ROH are congenital anomalies of the kidney and urinary tract [2]. ROH can lead to the development of pulmonary hypoplasia. Children with ROH tend to have elevated risk of respiratory complications and impaired lung function in infancy [3].

Most of the studies show serious consequences of this diagnosis when we include termination of pregnancy (TOP), survival rate and need for kidney replacement therapy [4–6]. Different practices in clinical centers and varying ethical beliefs influence the approach to managing this severe pathology; thus, no standardized management is currently available. Frequently, family counseling is solely carried out by obstetricians [7]*.* In some cases, multiprofessional teams of healthcare experts (e.g., a gynecologist, pediatric nephrologist, neonatologist, pediatric surgeon, and psychologist) have been employed to improve family counseling for affected families.

The present retrospective study aimed to evaluate ante-, peri-, and postnatal risk factors and the clinical outcome of 168 fetuses with prenatally diagnosed congenital kidney anomalies and different amniotic fluid levels, in a level I perinatal center. Our results will serve as a basis for improving the future counseling of affected families.

## Materials and methods

### Study population

This retrospective study included 168 pediatric patients who presented with congenital kidney anomalies from 2010–2019 at our level I perinatal center. On the basis of AF volume, the cohort was divided into three groups: normal amniotic fluid volume (NAF), low amniotic fluid volume (LAF), and ROH. ROH was sonographically defined as an amniotic fluid index (AFI) ≤ 5.1 cm and a single deepest amniotic fluid pocket (SDP) ≤ 2 cm. Previous studies have shown that these two measurement methods were valid for evaluating amniotic fluid in oligohydramnios [8, 9]. Patients with AFIs of 5.2 to 8 cm and an SDP of 2.1 to 4 cm were assigned to the LAF group. These three groups were evaluated for antenatal parameters and for perinatal and postnatal development with an elaborate case report form. Then, the three groups were compared with statistical analyses.

### Data collection

The collection of ultrasonographic images was performed by specialists in obstetrics. Local guidelines were applied for antenatal ultrasound screening in Germany. They provided three ultrasound examinations, one for each trimester [10]. The lowest AF measurement at any time during the pregnancy determined the assignment to one of the three AF groups. Prenatal information about each mother and child included prenatal suspected diagnoses, gestational week of diagnosis, sex of the fetus, maternal age, extrarenal manifestations, prenatal intervention methods, and whether the parents had decided to undergo TOP. Perinatal parameters were retrieved from natal registries. For all live-born children, data were recorded on the gestational week at birth, birth mode, APGAR scores at 5 min and 10 min, percentile of birth weight (calculated for German neonates according to [11]) and the need for neonatal intensive care. To compare outcomes among the three groups, data on live-born children at last examination were collected until 2019. The postnatal data acquisition included: postnatal diagnosis, calculated percentiles of weight (pediatric calculation for German children according to [12]), and a recent glomerular filtration rate (GFR). The GFR in children aged one year or older was estimated according to the cystatin C formula [13] for children with normal cystatin C laboratory control. Otherwise, GFR was calculated with a creatinine-based bedside formula, according to Schwartz et al. [14]. Additional follow-up parameters were: the number of hospitalizations, dialysis treatment, kidney transplantation, respiratory adaptation, feeding adaption, and time of death, when deceased.

### Statistical analysis

Initially, the parameters were analyzed with descriptive statistics. Categorial variables are expressed as the absolute and relative frequencies. Continuous, normally distributed data are expressed as the mean and standard deviation, and non-normally distributed data are expressed as the median and range. The relationship between two categorical variables was tested with the Chi-Square test. For continuous, non-normally distributed and ordinal variables, the Mann–Whitney U test was performed. The Kruskal–Wallis test was performed to compare the three groups: NAF, LAF, and ROH. Odds ratios (ORs) and 95% confidence intervals (CIs) were calculated for pre-and postnatal parameters to estimate the influence of ROH compared to the two other AF states. P-values < 0.05 were considered statistically significant. All statistical analyses were performed with SPSS Statistics 28.0 software for Mac (SPSS, Chicago, IL, USA).

## Results

### Prenatal period

**Fig. 1:**
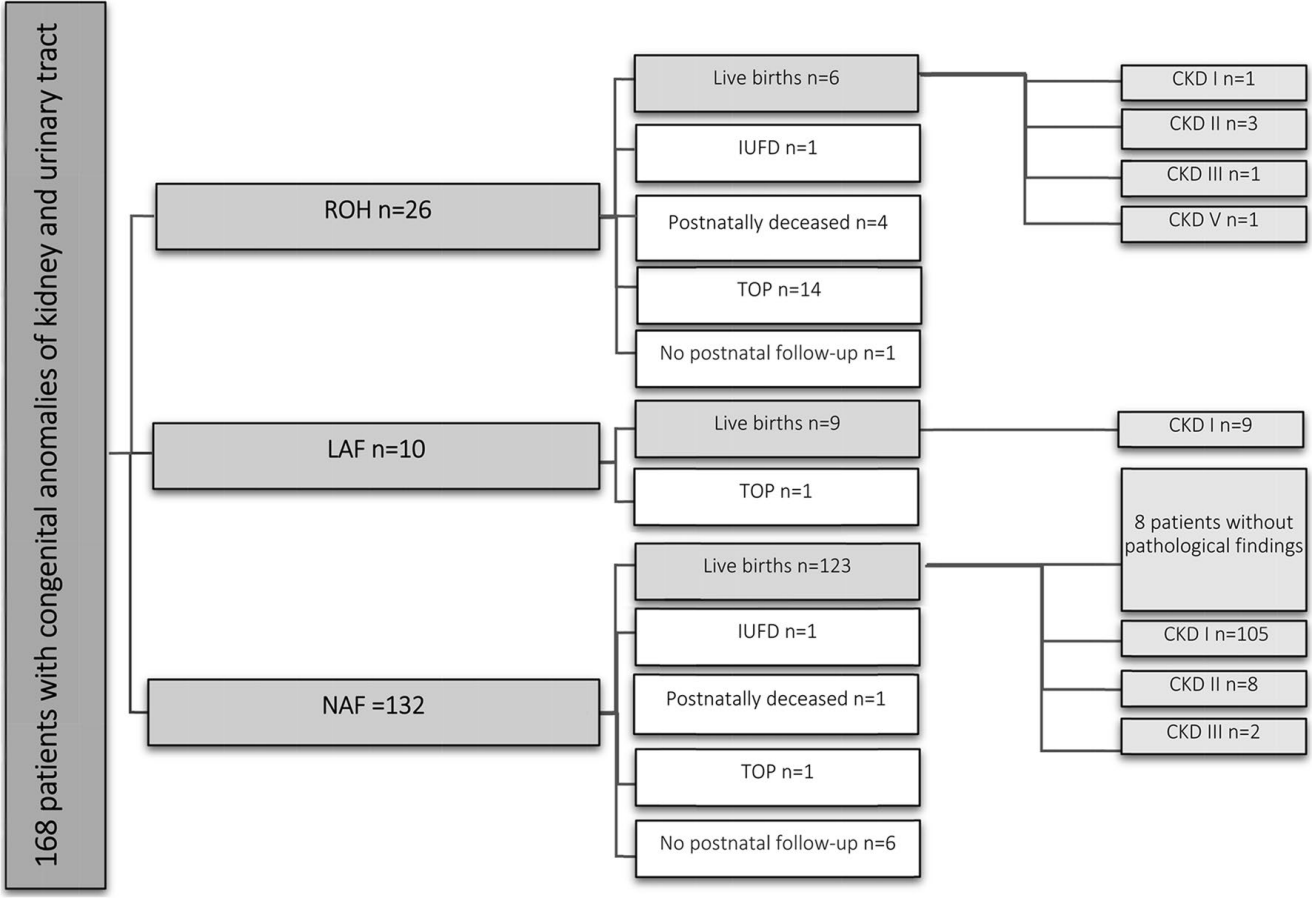
Outcomes in the three amniotic fluid groups. Patients with NAF and LAF showed significantly higher survival rates than children with ROH (*p* < 0.001). *ROH: renal oligohydramnios; LAF: low amniotic fluid volume; NAF: normal amniotic fluid volume; IUFD: intrauterine fetal death; TOP: termination of pregnancy; CKD: chronic kidney disease*

Among 168 fetuses with suspicious ultrasonographic diagnostic findings in the kidney and urinary tract, 26 (15.5%) displayed verified ROH, 132 (78.5%) displayed NAF, and 10 (6%) displayed LAF (Fig. 1). Based on ultrasonic examinations, 99 boys and 62 girls could be identified, but sex could not be determined in 7 children. The median follow-up for all patients was 636 days (range: 1–3148 days). In the ROH group, 14/26 (54%) of the parents chose a TOP, at a mean of 20 gestational weeks. Of the 14 TOPs performed, 10 had concomitant malformations. Among the children with ROH, the mother’s median age was 30 years (range: 19–38) (Table 1 and 2). ROH was diagnosed significantly earlier in the pregnancy (median gestational week 21) than either LAF (median gestational week 35) or NAF (median gestational week 32; *p* < 0.001). Three patients with ROH received a prenatal interventional treatment, either vesicoamniotic shunting (*n* = 1) or an amniotic fluid infusion (*n* = 2).

**Table 1:** Prenatal clinical data for the three amniotic fluid groups

Clinical data	ROH		LAF		NAF		*p* value
Number of patients (n)	26		10		132		
Sex (n)							
Male Female Not determined	11 (42.3) 11 (42.3) 4 (15.4)		6 (60) 4 (40) –		82 (62.1) 47 (35.6) 3 (2.3)		0.361
First diagnosed, week of gestation	21 (14–37)		35 (21–37)		32 (19–39)		< 0.001
ANH-classification APD (n) Normal Mild Moderate Severe Undefined	LK 4 (15.4) 1 (3.8) – – 21(80.8)	RK 5 (19.2) 1 (3.8) 1 (3.8) – 19 (73.1)	LK 3 (30) – 2 (20) 1 (10) 4 (40)	RK 2 (20) 1 (10) 1 (10) 1 (10) 5 (50)	LK 39 (29.5) 16 (12.1) 28 (21.2) 19 (14.4) 30 (22.7)	RK 41 (31.1) 15 (11.4) 30 (22.7) 14 (10.6) 32 (24.2)	
Mother’s age, years	30.0 (19–38)		26.5 (19–34)		29.0 (18–42)		0.396
Antenatal interventional treatment (n)	3 (11.5)		–		–		< 0.001

Data are the median (range) or number (%), as indicated. *ROH* renal oligohydramnios; *LAF* low amniotic fluid volume; *NAF* normal amniotic fluid volume; *ANH* antenatal hydronephrosis; *APD* anteroposterior diameter; *LK* left kidney; *RK* right kidney

**Table 2:** Perinatal and postnatal clinical data for the three amniotic fluid groups

Clinical data	ROH	LAF	NAF	*P* value
Children born alive (n)	10/26	9/10	124/132	
Birth, week of gestation	37 (22–38)	38 (36–40)	39 (28–42)	< 0.001
Birth mode (n)				
Natural Primary CS Secondary CS Emergency CS	4 (40) 3 (30) 1 (10) 2 (10)	8 (88.9) 1 (11.1) – –	90 (72.6) 16 (12.9) 12 (9.7) 6 (4.8)	< 0.001
APGAR score				
5 min 10 min	8 (0–9) 9 (0–10)	9 (7–10) 10 (8–10)	9 (0–10) 10 (0–10)	< 0.001 0.008
Neonatal intensive care^a^ (n)	8 (80)	4 (44.4)	33 (26.6)	< 0.001
Pulmonary hypoplasia (n)	3 (30)	–	2 (1.6)	< 0.001
Complicated feeding (n)	5 (50)	–		< 0.001
GFR (ml/min)	65 (7–112)	119 (98–114)	106 (40–207)	< 0.001
Dialysis + kidney transplantation (n)	1 (10)	–	–	< 0.001
Concomitant malformations (n)	2 (20)	1 (11)	4 (3.2)	< 0.001
Hospital admissions (n)	2 (0–25)	2 (0–20)	1 (0–16)	0.131

Data are the median (range) or number (%), as indicated. ^a^Admission to neonatal intensive care unit for mucus removal, intubation, mask ventilation, supplemental oxygen, buffering, or volume substitution. *ROH* renal oligohydramnios; *LAF* low amniotic fluid volume; *NAF* normal amniotic fluid volume; *CS* cesarian section; GFR: glomerular filtration rate

**Fig. 2:**
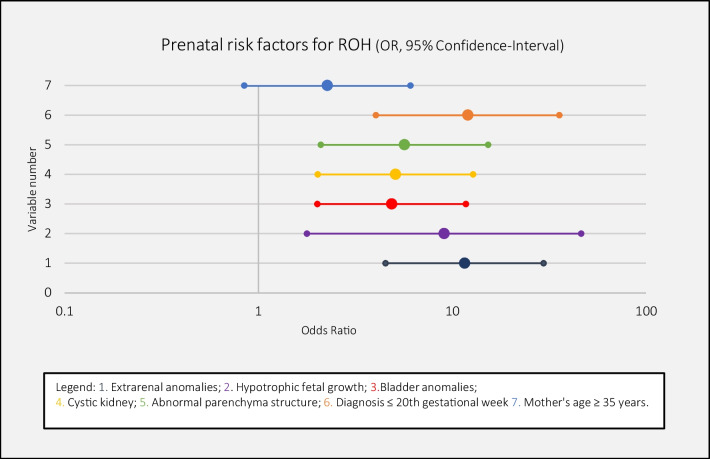
The odds of developing renal oligohydramnios (ROH), depending on the presence of various prenatal influencing factors. Symbols represent the odds ratios and lines indicate 95% confidence intervals

Among the prenatal sonographic patient diagnoses, cystic kidney malformations were predominant in the ROH group (30.8%). In contrast, the primary diagnoses in the NAF and LAF groups were vesicoureteral reflux (VUR) and hydronephrosis (NAF: 54.5%; LAF: 30%) as well as lower urinary tract obstruction for LAF 30% (Table 3). Sonographic measurements of anterior–posterior kidney pelvic diameters (APD) confirmed that antenatal hydronephrosis (ANH) was more common in NAF and LAF groups. Moreover, numerous factors were identified by ORs that influenced ROH development in children (Fig. 2). The parameters most highly associated with ROH development were extrarenal anomalies (OR: 11.6), a diagnosis prior to 20 weeks of gestation (OR: 12.1), and fetal growth disturbances (OR: 9.1). Extrarenal anomalies were defined as any additional genetic or structural anomaly present in other organs.

**Table 3:** Prenatal sonographic diagnoses for the three amniotic fluid groups

Diagnoses	ROH (*n* = 26)	LAF (*n* = 10)	NAF (*n* = 132)
Solitary functioning kidney	–	2 (20%)	25 (19%)
Duplex kidney, horseshoe kidney	1 (4%)	–	8 (6%)
Kidney dysplasia	2 (8%)	1 (10%)	2 (1.5%)
Kidney dystopia	–	–	6 (4.5%)
VUR and hydronephrosis	2 (8%)	3 (30%)	72 (55%)
LUTO	4 (15%)	3 (30%)	16 (12%)
ARPKD, ADPKD	8 (31%)	1 (10%)	–
Bilateral kidney agenesis	2 (8%) 4 (15%) *	–	–
Complex multiorgan disease	3 (11%)	–	3 (2%)

Data are the number (%). *ROH* renal oligohydramnios; *LAF* low amniotic fluid volume; *NAF* normal amniotic fluid volume; *VUR* vesicoureteral reflux; *LUTO* lower urinary tract obstruction; *TOP* termination of pregnancy; *ARPKD* autosomal recessive polycystic kidney disease; *ADPKD* autosomal dominant polycystic kidney disease

*Unilateral kidney agenesis and contralateral multicystic dysplastic kidney

### Perinatal period

From the perinatal period and later, only live-born children (*n* = 10/26 ROH, *n* = 124/132 NAF, *n* = 9/10 LAF) were included in the analyses (Table 2). Birth complications that led to an emergency cesarian section occurred significantly more frequently among children with ROH (*n* = 2/10), compared to those with LAF (*n* = 0/9) or NAF (*n* = 6/124; *p < *0.001). Moreover, children with ROH had significantly lower APGAR scores at 5 and 10 min (APGAR 5 min: 8, range: 0–9, *p* < 0.001; APGAR 10 min: 9, range: 0–10, *p* = 0.008). Finally, significantly more children with ROH required intensive neonatal care (*p* < 0.001) and intubation (*p* < 0.001). However, children in NAF and LAF groups also required intensive care in a considerable number of cases (LAF 44.4%, NAF 26.6%). The three groups received mask ventilation at similar frequencies: LAF: 1/9 (11.1%), NAF: 13/124 (10.5%), and ROH: 1/10 (10%). Comparing the pre-and postnatal diagnoses of the ROH group, the leading diagnosis changed from polycystic kidney disease (prenatal) to complex multiorgan disease (postnatal). In the NAF and LAF groups, the postnatal evaluation of the patient’s leading diagnosis did not differ from the prenatal sonographic evaluation.

### Mortality

An outcome analysis of the entire study cohort is shown in Fig. 1. In the ROH group, 10/26 children were born alive. Of these, 6/10 (60%) children survived the observation time. Four ROH patients died in the neonatal period, and of these, two children died due to a syndromic disease and two children died due to pulmonary hypoplasia (both with autosomal recessive kidney disease). In the NAF group, 1/124 children died due to VACTERL syndrome (i.e., vertebral defects, anal atresia, cardiac defects, tracheo-esophageal fistula, kidney anomalies, and limb abnormalities), with cystic kidney malformations and hydronephrosis (Table 4). Among the three groups, survival rates of children with NAF (*n* = 123/124) and LAF (*n* = 9/9) were significantly higher than that of children with ROH (*n* = 6/10; *p* < 0.001).

**Table 4:** Postnatal diagnoses, including autopsy data from terminated pregnancies

Diagnoses	ROH (*n* = 25, 1 lost to follow-up)	LAF (*n* = 10)	NAF (*n* = 126, 6 lost to follow-up)
Solitary functioning kidney	–	1 (10%)	25 (20%)
Duplex kidney, horseshoe kidney	1 (4%)	–	11 (9%)
Kidney dystopia	–	–	6 (5%)
VUR and hydronephrosis	–	4 (40%)	53 (42%)
LUTO	3 (12%) *(2 TOP)*	3 (30%)	15 (12%)
ARPKD, ADPKD	8 (32%) *(2 TOP, 1 IUFD, 2†)*	–	1 (1%)
Bilateral kidney agenesis	1 (4%) *(1 TOP)*	–	–
Complex multiorgan disease	12 (48%) *(9 TOP, 2†)*	2 (20%) *(1 TOP)*	7 (5%) *(1 TOP, 1 IUFD, 1 †)*
Normal finding	–		8 (6%)

Data are the number (%). *ROH* renal oligohydramnios; *LAF* low amniotic fluid volume; *NAF* normal amniotic fluid volume; *VUR* vesicoureteral reflux; *LUTO* lower urinary tract obstruction; *TOP* termination of pregnancy; *ARPKD* autosomal recessive polycystic kidney disease; *ADPKD* autosomal dominant polycystic kidney disease; *IUFD* intrauterine fetal death. *TOP* Termination of pregnancy; *†*: postnatally deceased

In the ROH group, three out of 26 children received therapeutic prenatal interventions. Of these children, one received a vesicoamniotic shunt placement and survived the observation time. Two children received AF infusions; of these, one died intrauterine and the other died in the neonatal period.

### Long-term outcome

We next evaluated the three groups (ROH, NAF, LAF) to investigate the relation of ROH on the postnatal development of a child diagnosed with a congenital kidney anomaly. We evaluated the weight percentiles for each of the 168 children at the time of the perinatal counseling conference, at birth, and at the last documented follow-up examination. The ROH group showed a significant drop in the median weight percentile from the prenatal period (48^th^ percentile, range 3–97), through the perinatal period (19^th^ percentile, range 3–97), and the postnatal period (3^rd^ percentile, range 3–75). In the two other groups, the weight percentiles remained relatively constant over the three time periods.

**Fig. 3:**
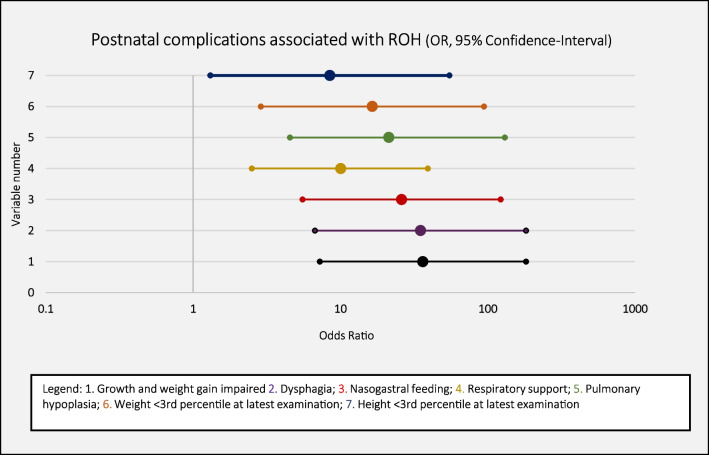
The odds of developing various postnatal complications in children with renal oligohydramnios (ROH). Symbols represent the odds ratios and lines indicate 95% confidence intervals

At the last examination, children with ROH presented significantly higher chronic kidney disease (CKD) stages than children in the two other groups (*p* = 0.044). The GFR was significantly lower in children with ROH (GFR: 65 ml/min) than in children with NAF (GFR: 106 ml/min) or LAF (GFR: 119 ml/min; *p* < 0.001). However, it should be emphasized that 5/6 children with ROH presented with CKD stages I–III at the last examination. One patient in the ROH group had CKD stage V and received consecutive peritoneal dialysis and a kidney transplantation. In comparison, none of the children in the NAF or LAF groups required kidney replacement therapy. Interestingly, the surviving children in the ROH group did not have significantly longer hospital stays or more surgeries than children with congenital kidney anomalies and either NAF or LAF (NAF: *p* = 0.131, LAF: *p* = 0.451). The most relevant differences in postnatal development between children with ROH compared to children with NAF or LAF were the risks of restricted growth and weight gain, respiratory issues, and feeding complications (Fig. 3).

## Discussion

Medical and technical progress has enabled accurate diagnostics in prenatal medicine. With the help of ultrasonographic fine diagnostics and fetal MRI, anomalies of the kidney and urinary tract can be well detected, but a detailed consultation is required for families about a child’s diagnosis and prognosis. The present study is one of the few to compare clinical data from the date of prenatal counseling through a postnatal follow-up among children with congenital kidney anomalies and different AF statuses. Other studies such as Mehler et al. and Zilberman et al. also compare different amniotic fluid levels but do not focus on a complete follow-up of all children [6, 15].

### Parental decision for TOP

In our ROH cohort, 54% of the parents chose a TOP, which limited the significance of our follow-up results. Other studies have shown lower termination rates, such as the 37% reported by Mehler et al. [6]. It must be emphasized that additional extrarenal malformations were observed in 93% of our TOP cohort, compared to only 53% (20/38) in the TOP cohort of Mehler et al. [6]. High TOP rates among patients with ROH also influenced the results of the PLUTO study (Percutaneous shunting in Lower Urinary Tract Obstruction); their high TOP rate eventually led to early study closure, due to lack of statistical power [16]. Families that were counseled by a local gynecologist and were later included in our study often had a pre-existing opinion about TOP. Independent of the diagnosis severity and ethical beliefs, we recommend effective communication between families, doctors, and professional multidisciplinary support personnel, and access to further testing and appointments [17].

### Survival

Survival rates of live-born children differed significantly among the AF groups. As shown in many previous studies, the lack of AF represented a poor prognostic factor for infant survival in the neonatal period [18–21]. In the present study, four children in the ROH group died in the neonatal period due to respiratory failure or syndromal disease. The survival rates of newborns with NAF and LAF were significantly higher than that of newborns with ROH. Like Krispin et al., we assumed that the survival and outcome of patients with intermediate oligohydramnios, represented by the LAF group, was similar to that observed among children with euhydramnios [22]. Both our NAF and LAF groups predominantly represented urinary flow disorders and LUTO – diagnoses with a good prognosis, when treated early. In contrast, newborns with ROH presented with a remarkable number of extrarenal anomalies, which were associated with multiorgan developmental complications.

We assumed that, once a patient with ROH surpassed the neonatal period, the mortality risk would be very low. This assumption was supported by the fact that 5/6 survivors had only mild to moderate CKD in infancy. Our study confirmed results shown in other studies [6, 20, 21] which showed that ROH was associated with a high neonatal mortality and morbidity. Nevertheless, the postnatal prognosis was favorable for children with ROH.

### Prognostic factors

Most pregnant women receive routine surveillance imaging during pregnancy. Consequently, kidney and urinary malformations may be discovered more frequently than before. As a result, caregivers must develop a comprehensive plan before birth [2]. In our study, we emphasized that multidisciplinary counseling must accompany the families, both in the antenatal setting and after birth, to develop a detailed follow-up program. The present study underlined the following facts associated with the development of ROH. In particular, patients who show antenatal malformations in the kidneys and urinary tract, paired with extrarenal anomalies, hypotrophic fetal growth, or a diagnosis of a kidney/urinary tract anomaly ≤ 20 gestational weeks, must draw the doctor’s attention to a possible underlying ROH. Our findings also emphasized that intrauterine growth retardation, detected with ultrasound, in association with ROH represents a poor prognosis, as shown previously by other research groups [23]. Moreover, our results supported the notion that, in particular, infants small for their gestational age that are born with ROH are at increased risk of death, compared to children with NAF [24]. In addition, extrarenal anomalies and an early diagnosis before 20 weeks of gestation are negative predictive factors for patients with ROH [6, 9, 25, 26].

We also showed that the emergency cesarean section and intubation rates in the ROH group were significantly higher than in the two other AF groups. At this point it should be added that children in the NAF and LAF groups are also prone to neonatal complications with subsequent need for intensive care (as shown in Table 2). Therefore, we suggest that delivery should be conducted in a center that provides a neonatal intensive care unit.

One of our most important results was that impaired growth and weight gain and nutritional uptake challenges were significantly higher in the ROH group compared to the NAF and LAF groups. All 168 patients in our cohort had prenatally diagnosed kidney and urinary tract anomalies, which can be associated with growth and weight issues. ROH and intrauterine growth retardation are two conditions that occur together, as many studies have already shown [27–30]. Our results underlined the notion that a lack of AF contributed even more to growth and weight gain issues. Due to these challenges, medical professionals should adjust treatment and postnatal care for fetuses with ROH as described by Zurowska et al. and Karlberg et al., for children with kidney failure and normal AF volumes [28, 31]. A paragraph of the Karlberg study makes the following statement about children with congenital kidney anomalies and growth impairment: “We speculate that the growth failure during fetal life and the first postnatal months reflects metabolic and/or nutritional influences and the impaired growth at 0.75–1.5 years of age is related to a partial insensitivity to growth hormone”. Based on our data we can state that growth and weight gain are associated with oligohydramnios. Our results also supported the conclusion that children with ROH had a higher long-term risk of respiratory complications, compared to children with NAF or LAF [3]. It is debatable whether restricted growth and weight gain, respiratory issues and feeding complications are independent or a sequence of events, since there were children in the ROH group without respiratory issues who still had feeding and growth complications.

These potential neonatal complications must be taken into consideration by doctors and families in prenatal care. The well-established guidelines in our center emphasized delivery and follow-up requisites for children with ROH. These requisites included the obligatory attendance of obstetricians, neonatologists, pediatric nephrologists, urologists, pulmonologists, and pediatric surgeons; the availability of the latest respiratory support system; and an intensive care unit.

### Study limitations

This study had some important limitations. First, the postnatal outcome data included only the patients who survived the neonatal period, which was a rather small patient cohort for the ROH group. Second, the three ROH children who received antenatal therapeutic interventions were not analyzed individually; hence, no firm conclusion could be drawn about the influence of antenatal interventions. Third, although we could draw a conclusion about the health issues associated with the presence of ROH, we did not analyze the quality of life of patients or their families. To achieve even better counseling for families affected by ROH, larger, multicenter prospective studies must be performed.

### Conclusion

In summary, this is one of the few studies to compare patients with anomalies of the kidney and urinary tract on the basis of AF volume with long-term follow-up. We identified several risk factors associated with the presence of ROH that should be focused on for appropriate family counseling. Our findings showed that a prenatal diagnosis of ROH was associated with elevated neonatal mortality compared to the mortality of children with normal or mildly reduced amounts of AF. Patients with ROH who survived the neonatal period had acceptable renal function without kidney replacement therapy. Not only the ROH group, but also the NAF and LAF groups have a significant number of pre-and postnatal complications.

### Supplementary Information

Below is the link to the electronic supplementary material.

Graphical Abstract (PPTX 52 KB)

## Data Availability

The datasets generated and analyzed during the current study are available from the corresponding author on reasonable request.
