# Supplementary material for: Amniotic fluid content in children with kidney and urinary tract anomalies determines pre- and postnatal development
Source: Pediatr Nephrol. 2023 May 23;38(11):3635–43. doi: 10.1007/s00467-023-05988-w (PMC10514154; doi:10.1007/s00467-023-05988-w)
Supplement: Supplementary file 1 — Graphical Abstract (PPTX 52 KB) [file 467_2023_5988_MOESM1_ESM.pptx]

## Slide 1
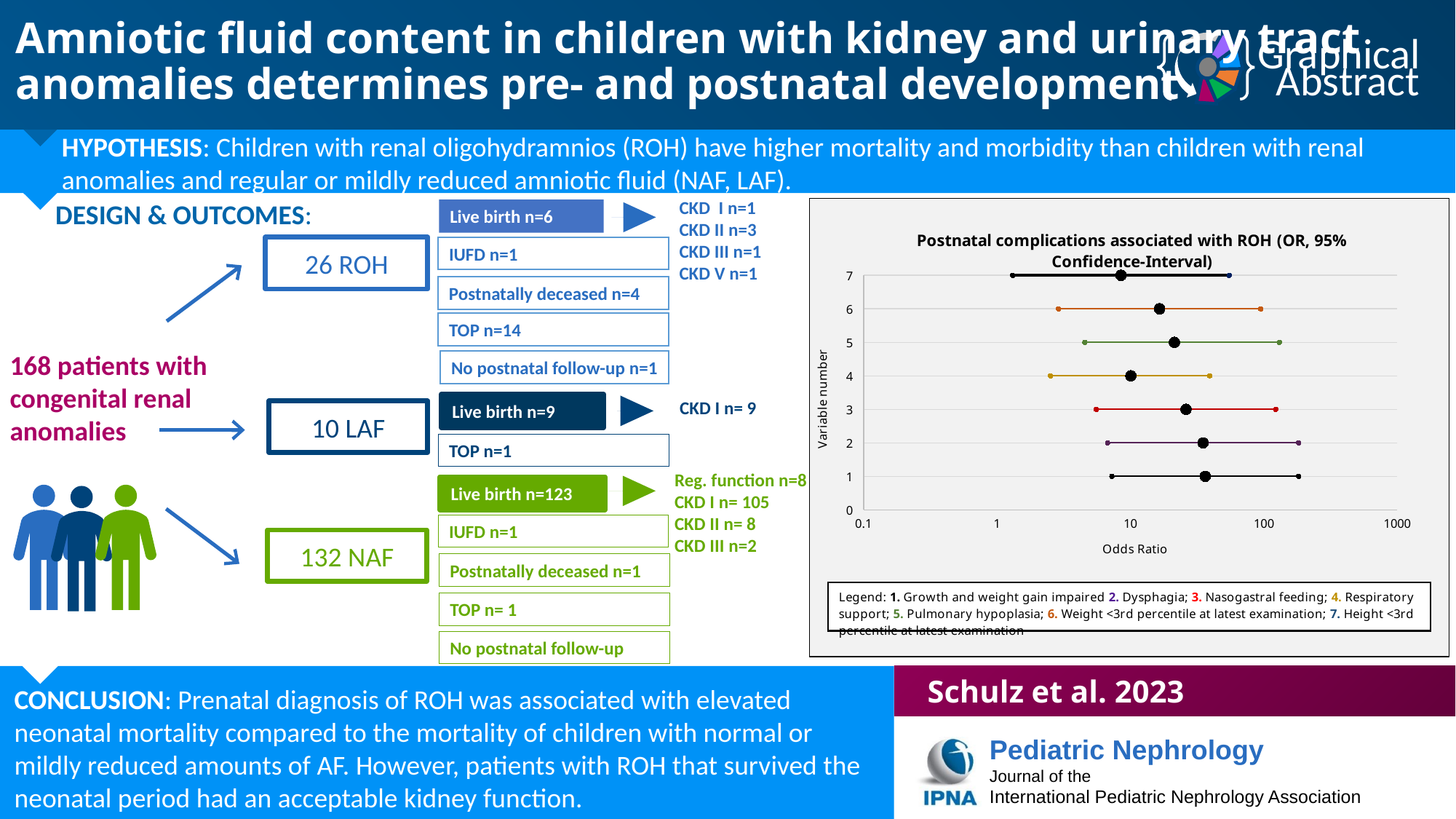

Amniotic fluid content in children with kidney and urinary tract
anomalies determines pre- and postnatal development
HYPOTHESIS: Children with renal oligohydramnios (ROH) have higher mortality and morbidity than children with renal anomalies and regular or mildly reduced amniotic fluid (NAF, LAF).
CKD I n=1
CKD II n=3
CKD III n=1
CKD V n=1
DESIGN & OUTCOMES:
### Chart: Postnatal complications associated with ROH (OR, 95% Confidence-Interval)
| Category | OR | Größe <3. Perzentile bei letzter U | Gewicht <3. Perzentile bei letzter U | Lungenhypoplasie | Respiratorischer Support | Nasogastrale Sonde | Schluckstörungen | Wachstum und Zunahme beeinträchtigt |
|---|---|---|---|---|---|---|---|---|Live birth n=6
IUFD n=1
26 ROH
Postnatally deceased n=4
TOP n=14
168 patients with congenital renal anomalies
No postnatal follow-up n=1
CKD I n= 9
Live birth n=9
10 LAF
TOP n=1
Reg. function n=8
CKD I n= 105
CKD II n= 8
CKD III n=2
Live birth n=123
IUFD n=1
132 NAF
Postnatally deceased n=1
TOP n= 1
No postnatal follow-up
Schulz et al. 2023
CONCLUSION: Prenatal diagnosis of ROH was associated with elevated neonatal mortality compared to the mortality of children with normal or mildly reduced amounts of AF. However, patients with ROH that survived the neonatal period had an acceptable kidney function.
.
